# Association of X-Ray Repair Cross-Complementing Group 1 Arg194Trp, Arg399Gln and Arg280His Polymorphisms with Head and Neck Cancer Susceptibility: A Meta-Analysis

**DOI:** 10.1371/journal.pone.0086798

**Published:** 2014-01-30

**Authors:** Wei Wu, Lu Liu, Zhihua Yin, Peng Guan, Xuelian Li, Baosen Zhou

**Affiliations:** 1 Department of Epidemiology, School of Public Health, China Medical University, Shenyang, China; 2 Key Laboratory of Cancer Etiology and Intervention, University of Liaoning Province, Shenyang, China; 3 Department of Preventive Dentistry, School of Stomatology, China Medical University, Shenyang, China; MD Anderson Cancer Center, United States of America

## Abstract

**Background:**

Previous studies on the association of X-ray repair cross-complementing group 1 (XRCC1) Arg194Trp, Arg399Gln, and Arg280His polymorphisms with head and neck cancer (HNC) have produced inconsistent results. The aim of the present study was to evaluate the effects of these three polymorphic variants on HNC risk.

**Methods:**

The PubMed and EMBASE databases were searched for genetic association studies on the XRCC1 Arg194Trp, Arg399Gln, and Arg280His polymorphisms and HNC risk. (The most recent search was conducted on 20 August, 2013.) Twenty-six studies were identified and meta-analysis was performed to evaluate the association between the polymorphism and HNC by calculating combined odds ratios and 95% confidence intervals.

**Results:**

No significant association was found under the allelic, homozygous, heterozygote, and dominant genetic models in the overall comparison. Further, no significant association between the XRCC1 Arg399Gln and Arg280His polymorphisms and HNC risk was detected under the four genetic models in subgroup analyses based on ethnicity, cancer site, and whether or not the studies had been adjusted for cigarette smoking and alcohol. However, in stratified analyses based on cancer site, a significant association was found between the XRCC1 Arg194Trp polymorphism and oral cancer under the allelic, heterozygote, and dominant models. The XRCC1 Arg194Trp polymorphism was significantly associated with HNC risk in studies that were adjusted for smoking and alcohol under the homozygous and heterozygote models.

**Conclusion:**

The meta-analysis results suggest that the XRCC1 Arg399Gln and Arg280His polymorphisms are probably not associated with the risk of HNC, but the XRCC1 Arg194Trp polymorphism was associated with increased risk of HNC in the subgroup analysis of studies adjusted for smoking and alcohol and with increased risk of oral cancer in the stratified analyses based on cancer site. Further studies with larger samples are needed to confirm these findings.

## Introduction

Head and neck cancer (HNC), including cancers in the oral cavity, pharynx (other than nasopharynx), and larynx, is the sixth most common cancer in the world [1]. Approximately 540,000 new cases and 271,000 deaths are reported annually worldwide, indicating a mortality of approximately 50% [2]. HNC is considered to be a complex disease because both genetic and environmental risk factors contribute to its etiology [3]. The principal risk factors for HNC include tobacco and alcohol use, and exposure to the human papillomavirus (HPV), which together contribute to the development of at least 90% of squamous cell carcinoma of the head and neck cases [1]. Furthermore, many recent studies have provided evidence that genetic factors including family history [4] and polymorphisms in genes [5]–[8] play important roles in the development of HNC.

Recent evidence indicates that DNA repair genes may determine individual susceptibility to HNC [9], [10]. Polymorphisms in the repair genes that encode enzymes may increase or decrease DNA repair capacity. The DNA repair pathway involves the direct reversal pathway, the excision repair pathway, and the post-replication/bypass pathway. The excision repair pathway includes base excision repair (BER), nucleotide excision repair, and mismatch repair [11]. X-ray repair cross-complementing group 1 (XRCC1) is an important DNA repair protein in the BER pathway [12]. In vitro and vivo studies have shown that XRCC1 plays a role either directly during the repair of single-strand breaks or indirectly during BER. Loss of XRCC1 activity resulted in decreased genetic stability, including the increased frequency of spontaneous and/or induced chromosome translocations and deletions [13]–[16]. Although more than 200 single nucleotide polymorphisms (SNPs) have been identified in XRCC1, only three common SNPs have been widely investigated in cancer risk. They are Arg194Trp (rs1799782), Arg280His (rs25489), and Arg399Gln (rs25487), located in exons 6, 9, and 10, respectively, of the XRCC1 gene [3]. In HapMap (http://snp.cshl.org/cgi-perl/gbrowse/hapmap24_B36/), the minor allele frequency (MAF) of Arg194Trp is 0.09 for Caucasians and 0.26 for Asians; the MAF of Arg399Gln is 0.37 for Caucasians and 0.26 for Asians; and the MAF of Arg280His is 0.04 for Caucasians and 0.09 for Asians. Some studies reported the association of the Arg194Trp [8], [17], Arg280His [17], [18], and Arg399Gln [19], [20] polymorphisms with risk of various cancers.

Lately, a number of studies have reported the association between XRCC1 polymorphisms and HNC risk, but the results are inconsistent. Tea et al. [9] and Ramachandran et al. [10] found that the Arg194Trp polymorphism might increase the HNC risk, while Matullo et al. [21] reported the opposite finding. The function of the Arg280His polymorphism is still not fully understood. Chuang et al. [22] conducted a pooled analysis and found that rare XRCC1 Arg280His homozygotes were associated with HNC risk after adjustment for cigarette smoking and alcohol consumption, while, in other studies, no such association was found [9], [23]–[25]. For the Arg399Gln polymorphism, the results are still controversial [10], [11], [24]. Therefore, we performed a meta-analysis to assess the association between the XRCC1 Arg194Trp, Arg280His and Arg399Gln polymorphisms and HNC risk.

## Materials and Methods

### Search Strategy

We searched PubMed and EMBASE databases for all genetic association studies on XRCC1 and HNC risk. (The most recent search was conducted on 20 August, 2013). Various combinations of the following terms were used in the search: “head and neck cancer”, “oral cancer”, “oropharyngeal cancer”, “laryngeal cancer”, “pharyngeal cancer”, “XRCC1”, “X-ray cross-complementing group 1”, “base excision repair”, “BER”, “SNP”, “single nucleotide polymorphism”, “polymorphism” and “variant”. Only English language papers were included in the search. The references cited in the original studies or review articles concerning the relevant topic were retrieved to potentially broaden the search for additional relevant publications.

### Inclusion and Exclusion Criteria

The following criteria were used to select the articles for the meta-analysis: (a) case-control study or cohort study methodology was used; (b) association of HNC with the XRCC1 Arg194Trp or Arg399Gln or Arg280His polymorphisms was explored; and (c) sufficient data of genotypes presented with estimated odds ratios (ORs) and 95% confidence intervals (CIs) were available. The exclusion criteria used were: (a) the control population included malignant tumor patients or the study had no controls; (b) insufficient information was available about genotype frequency or number; (c) duplicate publications or publications that contained overlapping data.

### Data Extraction

Information was extracted from all eligible publications carefully and independently by two investigators (Wei Wu and Lu Liu) using a standard protocol and data-collecting form based on the inclusion criteria. The original extraction data were checked by another investigator (Zhihua Yin), and disagreements were resolved by discussion among the three investigators. The following data were extracted: name of first author, year of publication, ethnicity of studied populations, site of cancer, genotyping method, source of controls, matching criteria, adjusted variables, and cases and controls with different genotypes.

### Statistical Analysis

The Hardy-Weinberg equilibrium (HWE) test [26] was conducted on the control groups to evaluate the genetic equilibrium of each study. A P value>0.05 was taken to indicate no significant disequilibrium. To avoid the inclusion of unknown heterogeneities, studies in which the distribution of the genotypes of the XRCC1 gene polymorphisms in the control groups not consistent with the HWE were excluded in the subsequent analysis. The MAF was computed in the control groups. MAF is an estimate of the frequency at which the less common allele occurs in a given population. The strength of the association between an XRCC1 polymorphism and HNC risk was assessed by combined odds ratios (ORs) with 95% confidence interval (CIs). The significance of the combined ORs was determined by a Z test and two-sided P values<0.05 were considered significant. The chi-square-based Q statistical test was used for heterogeneity analysis [27]. In this study, P values<0.05 were taken to indicate significant heterogeneity among studies. The random-effects model was used when heterogeneity was significant [28]; otherwise, the fixed-effects model was used [29]. Heterogeneity across studies was detected using an I^2^ test. I^2^ values of <25% were considered low, I^2^ values of 25% to 75% were considered moderate, and I^2^ values of >75% were considered high [30]. We calculated the OR using four different genetic models: allelic model (B vs. A), homozygous model (BB vs. AA), heterozygote model (AB vs. AA), and dominant model (BB+AB vs. AA), where A represents the major allele and B represents the minor allele. Stratified analyses of each study by ethnicity, cancer site, and whether the data had been adjusted for smoking and alcohol were also conducted using the four genetic models, to identify the relationship between the XRCC1 polymorphism and HNC risk. Whenever possible, adjusted ORs in a logistic model were used to compute combined OR and 95% CI for studies adjusted for smoking and alcohol. Furthermore, sensitivity analyses were conducted to confirm the stability and reliability of our results [31]. Visual inspection of Begg's funnel plot and Egger's test were used to evaluate the publication bias in the meta-analysis and P values<0.05 were considered statistically significant [32], [33]. All statistical tests were performed with the software Stata version 12.0 (Stata Corporation, College Station, TX, USA).

## Results

### Study Characteristics

**Figure 1:**
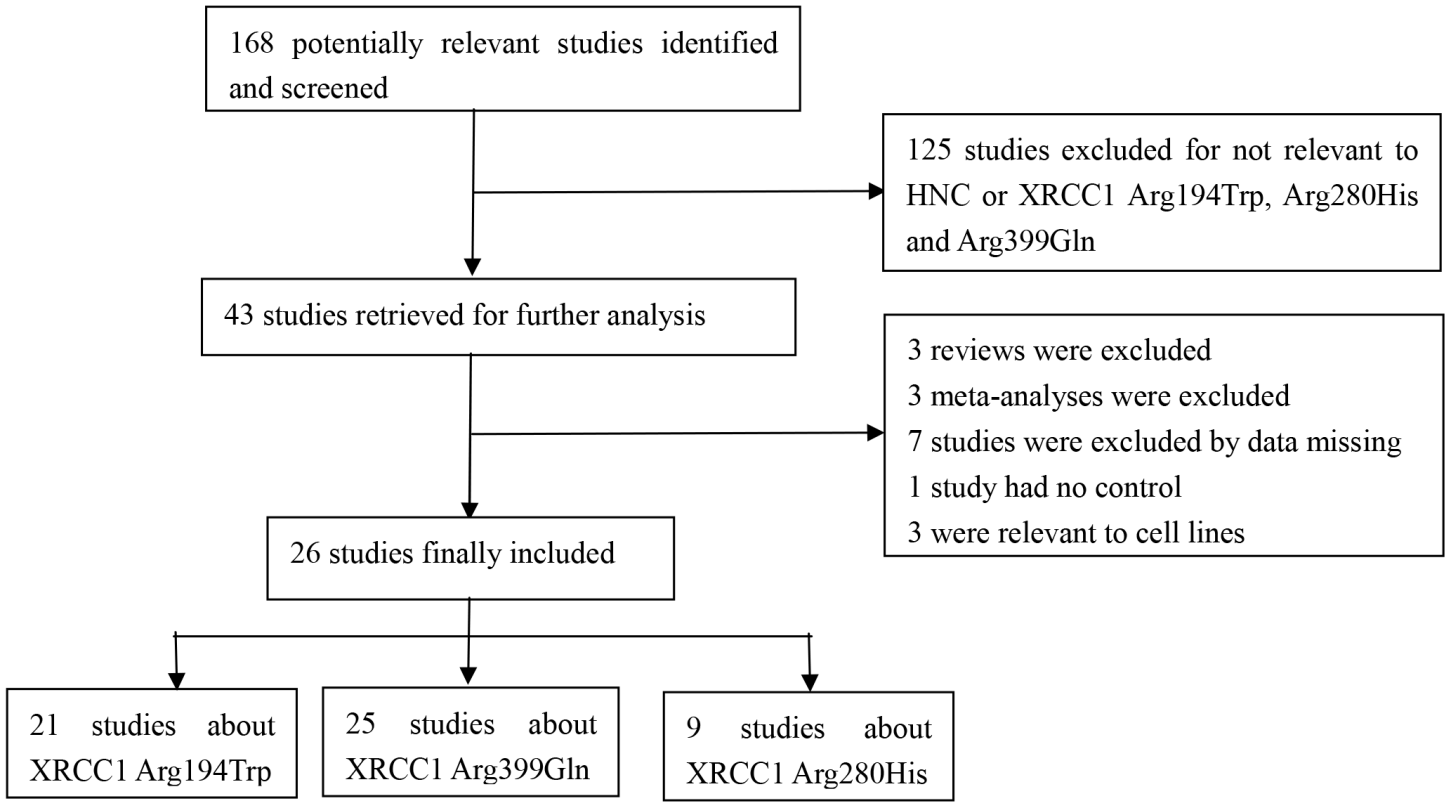
Flowchart of the process used for selection of eligible studies.

A total of 168 potentially relevant studies were retrieved after a comprehensive search of the PubMed and EMBASE databases (Figure 1), and 125 of these studies were excluded as not relevant to HNC or the XRCC1 Arg194Trp, Arg280His, and Arg399Gln polymorphisms. A further 17 studies were excluded including three reviews, three meta-analyses, seven studies with missing data, one study with no controls, and three studies relevant to cell lines. Consequently, 26 studies [9]–[11], [21], [23]–[25], [34]–[52] of the association of the XRCC1 Arg194Trp, Arg280His, and Arg399Gln polymorphisms with the risk of HNC were included in the meta-analysis. Twenty-one of the studies were about XRCC1 Arg194Trp, 25 were about XRCC1 Arg399Gln, and nine were about XRCC1 Arg280His (Figure 1).

The characteristics of the 26 eligible studies, including year of publication, ethnicity of studied populations, site of cancer, method of genotyping, source of controls, matching criteria, adjusted variables, cases and controls with different genotypes, HWE in controls, and MAF in controls for the XRCC1 Arg194Trp, Arg399Gln, and Arg280His polymorphisms are listed in Tables 1–3 respectively. All the studies were published between 1999 and 2013. The most commonly used genotyping method was polymerase chain reaction (PCR)-restriction fragment length polymorphism. The distribution of the genotypes of the XRCC1 Arg194Trp, Arg280His, and Arg399Gln polymorphisms in the control groups was consistent with the HWE, except in three of the studies. Two of these studies were related to the Arg194Trp polymorphism [37], [43], and one was related to the Arg399Gln polymorphism [51]. These studies were excluded from the subsequent analyses. Finally, to analyze the association of the three XRCC1 polymorphisms with the risk of HNC, 19 studies were selected for Arg194Trp, 24 studies were selected for Arg399Gln, and nine studies were selected for Arg280His.

**Table 1:** Characteristics of the Studies about the XRCC1 Arg194Trp polymorphism (rs1799782) Included in the Meta-analysis.

Author	Year	Ethnicity	Cancer Site	Genotyping Method	Control Source	Matching Criteria	Adjusted Variables	Case				Control				HWE^d^	MAF^e^
								N	AA	AB	BB	N	AA	AB	BB		
Dos Reis	2013	Mixed	Oral	PCR-RFLP	PB^a^	Age, sex, race, smoke	None	150	127	23	0	150	123	24	3	0.174	0.100
Yen	2008	Asian	Oral	PCR-RFLP	PB	None	Age, sex, alcohol, smoke, betel nut chewing	103	48	40	15	98	54	35	9	0.348	0.270
Sturgis	1999	Mixed	Head&neck	PCR-RFLP	HB^b^	Age, sex, race	Age, sex, ethnicity, smoke, alcohol	203	180	22	1	424	363	61	0	0.110	0.072
Majumder	2005	Asian	Oral	PCR-RFLP	HB	None	None	310	249	58	3	348	285	57	6	0.122	0.099
Harth	2008	Caucasian	Head&neck	RT-PCR	HB	None	Age, sex	258	217	40	1	300	259	39	2	0.690	0.072
Applebaum	2009	Mixed	Head&neck	PCR-RFLP	PB	Age, sex	Age, sex, race, education, smoke, alcohol, HPV16	484	427	55	2	549	485	61	3	0.476	0.061
Majumder	2007	Asian	Oral	PCR-RFLP	HB	None	Age, sex, smoke	309	248	58	3	387	317	62	8	0.022	0.101
Tae	2004	Asian	Head&neck	MAPA	HB	None	Age, smoke, alcohol	120	59	52	9	145	101	39	5	0.611	0.169
Varzim	2003	Caucasian	Larynx	PCR-RFLP	PB	None	None	88	80	8	0	178	160	18	0	0.477	0.051
Csejtei	2009	Caucasian	Head&neck	PCR-RFLP	HB	Age, sex	None	108	96	11	1	102	85	15	2	0.191	0.093
Kowalski	2009	Caucasian	Head&neck	PCR-RFLP	HB	Age	None	92	71	21	0	124	102	22	0	0.278	0.089
Demokan	2005	Caucasian	Head&neck	PCR-RFLP	PB	None	None	95	78	14	3	98	88	8	2	0.004	0.061
Ramachandran	2006	Asian	Oral	PCR-RFLP	PB	Age, sex	Age, sex, smoke, alcohol, betel quid chewing	110	66	37	7	110	90	19	1	0.998	0.095
Olshan	2002	Caucasian	Head&neck	PCR-RFLP	HB	Age, sex	Age, sex	98	82	16	0	161	135	26	0	0.265	0.081
Kietthubthew	2006	Asian	Oral	PCR-RFLP	PB	Age, sex, smoke, alcohol, betel chewing, religion	Other genes, betel quid chewing	106	40	50	16	164	77	67	20	0.365	0.326
Kumar	2012	Asian	Head&neck	PCR-RFLP	PB	Age, sex, habits	None	278	144	111	23	278	121	131	26	0.264	0.329
Gugatschka	2011	Caucasian	Head&neck	TaqMan	PB	None	None	168	148	20	0	463	397	63	3	0.772	0.075
Ho	2007	Mixed	Oral	PCR-RFLP	HB	None	Age, sex, ethnicity, family history, smoke, alcohol, radiation exposure	137	108	29	0	503	433	69	1	0.306	0.071
Rydzanicz	2005	Caucasian	Head&neck	PCR-RFLP	PB	Smoke, occupational exposure	None	182	165	16	1	143	129	14	0	0.538	0.049
Gajecka	2005	Caucasian	Larynx	PCR-RFLP	PB	None	None	290	262	27	1	325	291	33	1	0.950	0.054
Matullo	2006	Caucasian	Head&neck	TaqMan	UK^c^	None	None	82	78	4	0	1094	951	141	2	0.171	0.066

a PB: Population based;

b HB: Hospital based;

c UK: Unknown or unstated;

d HWE: Hardy-Weinberg equilibrium in controls;

e MAF: Minor allele frequency in controls.

**Table 2:** Characteristics of the Studies about the XRCC1 Arg399Gln polymorphism (rs25478) Included in the Meta-analysis.

Author	Year	Ethnicity	Cancer Site	Genotyping Method	Control Source	Matching Criteria	Adjusted Variables	Case				Control				HWE^d^	MAF^e^
								N	AA	AB	BB	N	AA	AB	BB		
Dos Reis	2013	Mixed	Oral	PCR-RFLP	PB^a^	Age, sex, race, smoke	None	150	64	62	24	150	62	54	34	0.002	0.407
Yuan	2012	Asian	Head&neck	TaqMan	PB	Age, sex	Age, sex, smoke, alcohol	390	221	146	23	886	481	339	66	0.558	0.266
Sturgis	1999	Mixed	Head&neck	PCR-RFLP	HB^b^	Age, sex, race	None	203	94	77	32	424	181	197	46	0.483	0.341
Majumder	2005	Asian	Oral	PCR-RFLP	HB	None	None	310	135	143	32	348	158	163	27	0.088	0.312
Harth	2008	Caucasian	Head&neck	RT-PCR	HB	None	Age, sex	310	114	166	30	300	143	121	36	0.189	0.322
Applebaum	2009	Mixed	Head&neck	PCR-RFLP	PB	Age, sex	Age, sex, race, education, smoke, alcohol, HPV16	483	192	229	62	547	232	246	69	0.763	0.351
Li	2007	Caucasian	Head&neck	PCR-RFLP	PB	Age, sex, race	Age, sex, smoke, alcohol	830	335	374	121	854	360	385	109	0.702	0.353
Majumder	2007	Asian	Oral	PCR-RFLP	HB	None	Age, sex, smoke	309	134	143	32	385	170	179	36	0.255	0.326
Tae	2004	Asian	Head&neck	MAPA	HB	None	Age, smoke, alcohol	129	69	51	9	157	86	64	7	0.251	0.248
Varzim	2003	Caucasian	Larynx	PCR-RFLP	PB	None	None	88	37	40	11	178	80	80	18	0.759	0.326
Csejtei	2009	Caucasian	Head&neck	PCR-RFLP	HB	Age, sex	None	108	50	47	11	102	53	41	8	0.986	0.279
Kowalski	2009	Caucasian	Head&neck	PCR-RFLP	HB	Age	None	92	37	44	11	124	49	53	22	0.253	0.391
Demokan	2005	Caucasian	Head&neck	PCR-RFLP	PB	None	None	95	42	41	12	98	39	46	13	0.922	0.367
Krupa	2011	Caucasian	Larynx	PCR-RFLP	HB	Age, sex	None	253	93	111	49	253	105	113	35	0.603	0.362
Kostrzewska-Poczekaj	2013	Caucasian	Head&neck	PCR-RFLP	PB	None	None	290	110	154	26	158	50	81	27	0.550	0.427
Jelonek	2010	Caucasian	Head&neck	PCR-RFLP	PB	Age, sex	None	104	47	50	7	110	35	62	13	0.068	0.400
Ramachandran	2006	Asian	Oral	PCR-RFLP	PB	Age, sex	Age, sex, smoke, alcohol, betel quid chewing	110	46	48	16	110	73	33	4	0.910	0.186
Olshan	2002	Caucasian	Head&neck	PCR-RFLP	HB	Age, sex	Age, sex	98	45	50	3	161	62	82	17	0.183	0.360
Kietthubthew	2006	Asian	Oral	PCR-RFLP	PB	Age, sex, smoke, alcohol, betel chewing, religion	None	106	55	45	6	164	67	74	23	0.724	0.366
Kumar	2012	Asian	Head&neck	PCR-RFLP	PB	Age, sex, habits	None	278	128	124	26	278	98	144	36	0.133	0.388
Gugatschka	2011	Caucasian	Head&neck	TaqMan	PB	None	None	168	70	74	24	463	204	198	61	0.241	0.346
Ho	2007	Mixed	Oral	PCR-RFLP	HB	None	Age, sex, ethnicity, family history, smoke, alcohol, radiation exposure	138	61	62	15	503	220	216	67	0.229	0.348
Rydzanicz	2005	Caucasian	Head&neck	PCR-RFLP	PB	Smoke, occupational exposure	None	182	63	98	21	143	59	63	21	0.535	0.367
Gajecka	2005	Caucasian	Larynx	PCR-RFLP	PB	None	None	293	106	153	34	319	124	145	50	0.484	0.384
Matullo	2006	Caucasian	Head&neck	TaqMan	UK^c^	None	None	82	34	38	10	1094	484	482	128	0.632	0.337

a PB: Population based;

b HB: Hospital based;

c UK: Unknown or unstated;

d HWE: Hardy-Weinberg equilibrium in controls;

e MAF: Minor allele frequency in controls.

**Table 3:** Characteristics of the Studies about the XRCC1 Arg280His polymorphism (rs25489) Included in the Meta-analysis.

Author	Year	Ethnicity	Cancer Site	Genotyping Method	Control Source	Matching Criteria	Adjusted Variables	Case				Control				HWE^c^	MAF^d^
								N	AA	AB	BB	N	AA	AB	BB		
Majumder	2005	Asian	Oral	PCR-RFLP	HB^a^	None	None	310	228	79	3	348	264	81	3	0.232	0.125
Harth	2008	Caucasian	Head&neck	RT-PCR	HB	None	Age, sex	312	283	28	1	300	270	30	0	0.362	0.050
Applebaum	2009	Mixed	Head&neck	PCR-RFLP	PB^b^	Age, sex	Age, sex, race, education, smoke, alcohol, HPV16	484	437	46	1	548	492	52	4	0.052	0.055
Majumder	2007	Asian	Oral	PCR-RFLP	HB	None	Age, sex, smoke	307	225	79	3	387	297	87	3	0.213	0.120
Tae	2004	Asian	Head&neck	MAPA	HB	None	Age, smoke, alcohol	135	113	21	1	168	139	29	0	0.221	0.086
Ramachandran	2006	Asian	Oral	PCR-RFLP	PB	Age, sex	Age, sex, smoke, alcohol, betel quid chewing	110	77	31	2	110	83	26	1	0.502	0.127
Kumar	2012	Asian	Head&neck	PCR-RFLP	PB	Age, sex, habits	None	278	129	123	26	278	142	116	20	0.575	0.281
Gugatschka	2011	Caucasian	Head&neck	TaqMan	PB	None	None	168	159	9	0	463	430	32	1	0.621	0.037
Ho	2007	Mixed	Oral	PCR-RFLP	HB	None	Age, sex, ethnicity, family history, smoke, alcohol, radiation exposure	138	125	13	0	503	453	50	0	0.241	0.050

a HB: Hospital based;

b PB: Population based;

c HWE: Hardy-Weinberg equilibrium in controls;

d MAF: Minor allele frequency in controls.

### Quantitative Data Synthesis

**Figure 2:**
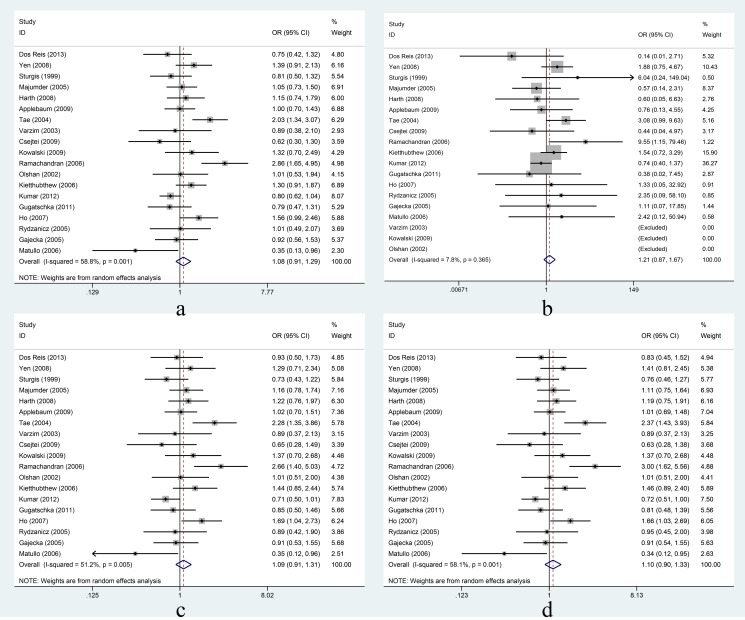
Association between XRCC1 Arg194Trp and risk of head and neck cancer under four genetic models. Forest plots for a: Trp vs. Arg; b: TrpTrp vs. ArgArg; c: ArgTrp vs. ArgArg; d: TrpTrp+ ArgTrp vs. ArgArg. Random-effects models were used for a, c, and d; a fixed-effects model was used for b. Squares and horizontal lines represent the study-specific OR and 95% CI respectively; diamond indicates the summary OR and 95% CI.

XRCC1 Arg194Trp: In the overall comparison, the Arg194Trp polymorphism was not significantly associated with HNC risk under the four different genetic models (Figure 2). Further, in the subgroup analyses based on ethnicity, the Arg194Trp polymorphism was found to be a risk factor in Asians while it was a protective factor in Caucasians under all genetic models; however, the association of the Arg194Trp polymorphism with HNC risk in Asians and in Caucasians was not significant (Table 4). In the stratified analyses based on cancer site, the Arg194Trp polymorphism was significantly associated with oral cancer using the allelic, heterozygote, and dominant models (allelic model: OR = 1.35, 95%CI = 1.00–1.82, I^2^ = 63.5%, *P_heterogeneity_* = 0.02; heterozygote model: OR = 1.40, 95%CI = 1.13–1.73, I^2^ = 28.5%, *P_heterogeneity_* = 0.22; dominant model: OR = 1.40, 95%CI = 1.14–1.72, I^2^ = 53.1%, *P_heterogeneity_* = 0.06), but not significantly associated with oral cancer using the homozygous model. The Arg194Trp polymorphism was not significantly associated with larynx cancer under all four genetic models (Table 4). In the analyses of studies adjusted for smoking and alcohol, the Arg194Trp polymorphism was significantly associated with HNC risk under the homozygous and heterozygote models (homozygous model: OR = 2.21, 95%CI = 1.44–3.38, I^2^ = 0.0%, P_heterogeneity_ = 0.50; heterozygote model: OR = 1.65, 95%CI = 1.15–2.38, I^2^ = 50.0%, P_heterogeneity_ = 0.01), but the association was not significant under the dominant model. When the studies were not adjusted for smoking and alcohol, the Arg194Trp polymorphism was not significantly associated with HNC risk using any of the four genetic models (Table 4).

**Table 4:** Stratified analyses of the association of the XRCC1 Arg194Trp (rs1799782), XRCC1 Arg399Gln (rs25487), and XRCC1 Arg280His (rs25489) polymorphisms with HNC risk.

XRCC1(polymorphism)	Variables	N^a^	B vs. A			BB vs. AA			AB vs. AA			BB+AB vs. AA		
			OR(95%CI)	*P* b	I^2^ (%)	OR(95%CI)	*P*	I^2^ (%)	OR(95%CI)	*P*	I^2^ (%)	OR(95%CI)	*P*	I^2^ (%)
XRCC1 Arg194Trp	Ethnicity													
(rs1799782)	Asian	6	1.39^c^(0.97–1.98)	0.00	81.2	1.34(0.93–1.92)	0.052	54.5	1.40^c^(0.93–2.10)	0.00	75.9	1.46^c^(0.95–2.24)	0.00	80.4
	Caucasian	9	0.90(0.74–1.11)	0.50	0.0	0.78(0.27–2.30)	0.91	0.0	0.92(0.74–1.14)	0.52	0.0	0.91(0.74–1.12)	0.49	0.0
	Cancer Site													
	Oral	6	1.35^c^(1.00–1.82)*	0.02	63.5	1.48(0.92–2.38)	0.19	33.0	1.40(1.13–1.73)*	0.22	28.5	1.40(1.14–1.72)*	0.06	53.1
	Larynx	2	0.92(0.59–1.42)	0.95	0.0	1.11(0.07–17.85)	N/A	N/A	0.90(0.57–1.43)	0.97	0.0	0.98(0.58–1.43)	0.96	0.0
	Smoking and alcohol													
	Adjusted	6	N/A	N/A	N/A	2.21(1.44–3.38)*	0.50	0.0	1.65^c^(1.15–2.38)*	0.01	50.0	1.41^c^(0.42–4.71)	0.00	90.4
	Unadjusted	13	0.93(0.81–1.06)	0.37	7.5	0.92(0.61–1.38)	0.77	0.0	0.93(0.80–1.08)	0.39	5.2	1.07^c^(0.89–1.29)	0.01	49.0
XRCC1 Arg399Gln	Ethnicity													
(rs25487)	Asian	7	1.02^c^(0.80–1.29)	0.00	80.3	1.03^c^(0.62–1.71)	0.00	73.6	0.98^c^(0.78–1.22)	0.03	58.5	1.00^c^(0.76–1.30)	0.00	74.3
	Caucasian	14	1.02(0.95–1.10)	0.09	35.9	0.98(0.84–1.15)	0.06	40.4	1.10(0.99–1.23)	0.24	19.6	1.07(0.98–1.19)	0.16	27.2
	Cancer Site													
	Oral	5	1.09^c^(0.80–1.49)	0.00	82.3	1.14^c^(0.60–2.19)	0.00	76.1	1.06(0.89–1.27)	0.06	55.5	1.11^c^(0.79–1.57)	0.00	74.7
	Larynx	3	1.09(0.94–1.28)	0.37	0.2	1.14(0.82–1.59)	0.17	44.4	1.16(0.92–1.46)	0.89	0.0	1.16(0.93–1.44)	0.94	0.0
	Smoking and alcohol													
	Adjusted	6	N/A	N/A	N/A	1.13^c^(0.81–1.56)	0.04	56.5	1.07(0.94–1.21)	0.13	41.2	1.01(0.88–1.16)	0.72	0.0
	Unadjusted	18	0.96^c^(0.88–1.06)	0.02	46.4	0.90^c^(0.72–1.12)	0.02	46.7	1.01^c^(0.88–1.15)	0.04	39.7	1.03^c^(0.90–1.18)	0.00	55.8
XRCC1 Arg280His	Ethnicity													
(rs25489)	Asian	5	1.16(0.99–1.35)	0.97	0.0	1.46(0.86–2.47)	0.97	0.0	1.15(0.96–1.38)	0.93	0.0	1.17(0.98–1.40)	0.94	0.0
	Caucasian	2	0.87(0.57–1.33)	0.54	0.0	1.66(0.20–13.63)	0.62	0.0	0.84(0.54–1.31)	0.74	0.0	0.85(0.55–1.32)	0.64	0.0
	Cancer Site													
	Oral	4	1.14(0.94–1.39)	0.89	0.0	1.38(0.49–3.83)	0.91	0.0	1.15(0.93–1.43)	0.91	0.0	1.16(0.93–1.43)	0.89	0.0
	Smoking and alcohol													
	Adjusted	4	N/A	N/A	N/A	1.59(0.08–32.17)	0.02	82.5	1.22(0.91–1.63)	0.10	51.7	0.98(0.52–1.86)	N/A	N/A
	Unadjusted	5	1.11(0.95–1.30)	0.75	0.0	1.43(0.84–2.43)	0.98	0.0	1.10(0.92–1.33)	0.76	0.0	1.09(0.93–1.27)	0.86	0.0

a Number of comparisons;

b P-value for Q-test;

c The random-effects model was used when the P-value for the Q-test for heterogeneity was <0.05, otherwise the fixed-effects model was used.

* Statistically significant, P<0.05.

**Figure 3:**
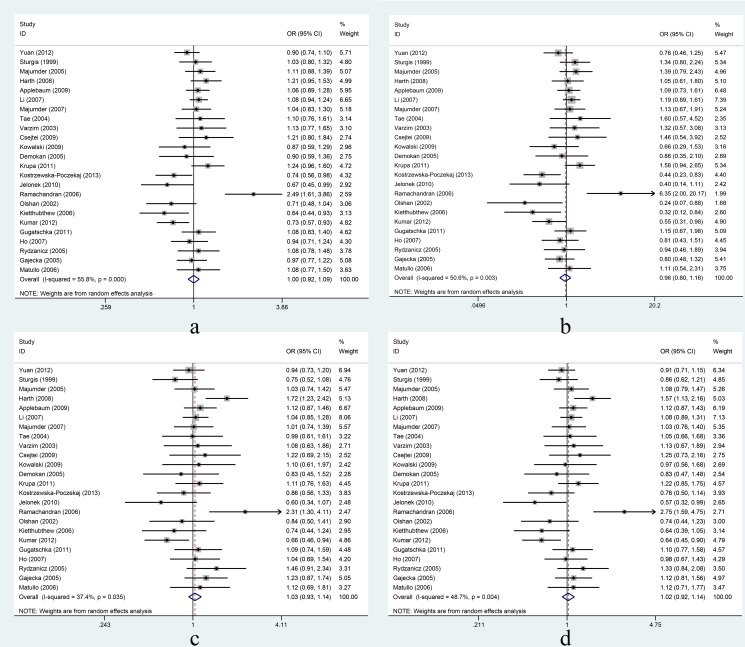
Association between XRCC1 Arg399Gln and risk of head and neck cancer under four genetic models. Forest plots for a: Gln vs. Arg; b: GlnGln vs. ArgArg; c: ArgGln vs. ArgArg; d: GlnGln+ ArgGln vs. ArgArg. Random-effects models were used for c and d; fixed-effects models were used for a and b. Squares and horizontal lines represent the study-specific OR and 95% CI respectively; diamond indicates the summary OR and 95% CI.

XRCC1 Arg399Gln: In the overall comparison, the Arg399Gln polymorphism was not significantly associated with HNC risk under the four different genetic models (Figure 3). Further, in the subgroup analyses based on ethnicity, the Arg399Gln polymorphism was not significantly associated with HNC risk in Asians or Caucasians (Table 4). In the stratified analyses based on cancer site, the Arg399Gln polymorphism was not significantly associated with oral cancer or larynx cancer (Table 4). When studies either adjusted or unadjusted for smoking and alcohol were analyzed, the Arg399Gln polymorphism was not significantly associated with HNC risk (Table 4).

**Figure 4:**
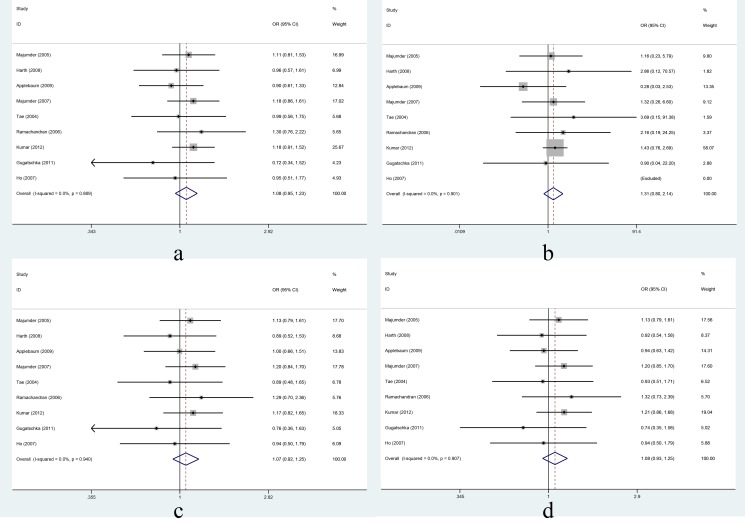
Association between XRCC1 Arg280His and risk of head and neck cancer under four genetic models. Forest plots for a: His vs. Arg; b: HisHis vs. ArgArg; c: ArgHis vs. ArgArg; d: HisHis+ ArgHis vs. ArgArg. Fixed-effects models were used for a, b, c, and d. Squares and horizontal lines represent the study-specific OR and 95% CI respectively; diamond indicates the summary OR and 95% CI.

XRCC1 Arg280His: In the overall comparison, the Arg280His polymorphism was not significantly associated with HNC risk under the four different genetic models (Figure 4). Further, in the subgroup analyses based on ethnicity, the Arg280His polymorphism was not significantly associated with HNC risk in Asians or Caucasians (Table 4). In the stratified analyses based on cancer site, the Arg280His polymorphism was not significantly associated with oral cancer (Table 4). When studies either adjusted or unadjusted for smoking and alcohol were analyzed, the Arg280His polymorphism was not significantly associated with HNC risk (Table 4).

### Heterogeneity Analysis

Evidence of heterogeneity between studies in this meta-analysis was detected for XRCC1 Arg194Trp, and Arg399Gln, but the reasons for the heterogeneity were unclear. In the subgroup analyses, significant heterogeneity was found in the studies that used Asian populations, but not in the studies that used Caucasians, indicating that the publications that used Asians were probably the main source of heterogeneity in our study. In addition, significant heterogeneity was found in studies among oral cancers but not larynx cancers, indicating that the publications that focused on oral cancers were another probable source of heterogeneity. HNC includes cancers from different sites and risk factors for these cancers are different. Therefore, further studies with larger sample sizes and different tumor sites are needed to investigate the possible sources of the heterogeneity.

### Sensitivity Analysis

Sensitivity analyses were conducted to assess the influence of individual studies on the combined ORs by omitting each study in turn. For all three polymorphisms under all four genetic models, the significance of the combined ORs was not materially altered by the exclusion of any individual study (data not shown). This result indicated that our results were statistically robust. Figure S1 shows the sensitivity analysis of XRCC1 Arg194Trp obtained under the allelic model by deleting of one study at a time.

### Publication Bias

Begg's funnel plot and Egger's test were used to estimate the publication bias in the literature. For all three polymorphisms, the shapes of the Begg's funnel plots under all four genetic models showed no obvious asymmetry. Figure S2 shows the shape of the Begg's funnel plot for XRCC1 Arg399Gln under the dominant model. Egger's test also did not reveal significant evidence of publication bias for the three polymorphisms under all four genetic models (data not shown); the one exception was for XRCC1 Arg280His under the heterozygote model (t = −2.56, P = 0.037). Nevertheless, we found no significant difference between the corrected OR and uncorrected OR in the trim and fill analysis, which supported the robustness of our findings.

## Discussion

In the overall comparison, the meta-analysis detected no significant association between the XRCC1 Arg194Trp, Arg399Gln, and Arg280His polymorphisms and HNC risk under all four genetic models. Further, in the subgroup analyses based on ethnicity, cancer site, and whether adjusted or unadjusted for smoking and alcohol, no significant association was found between the XRCC1 Arg399Gln, and Arg280His polymorphisms and HNC risk under the four genetic models. Nevertheless, in the stratified analyses based on cancer site, significant association was found between the XRCC1 Arg194Trp polymorphism and oral cancer under the allelic, heterozygote, and dominant models. When the studies adjusted for smoking and alcohol were analyzed, significant association was found between the XRCC1 Arg194Trp polymorphism and HNC risk under the homozygous and heterozygote models. Our results indicated that, while the XRCC1 Arg399Gln and Arg280His polymorphisms may not increase or decrease the risk of HNC, when cigarette smoking and alcohol consumption were taken into account, the XRCC1 Arg194Trp polymorphism was associated with increased risk of HNC and also may modulate genetic susceptibility to oral cancer.

The XRCC1Arg194Trp polymorphism is located in the region of the protein that separates the DNA polymerase-b and poly (ADP-ribose) polymerase-interacting domains. Tae et al. reported a highly significant association under the dominant genetic model of XRCC1 Arg194Trp with increased risk of squamous cell carcinoma of the head and neck among Korean patients and normal controls [9]. However, most other studies have found no association of XRCC1 Arg194Trp with HNC risk [11], [24], [25], [34], [35], [40], [46], [48]. In the present study, an intriguing finding was that the Arg194Trp polymorphism was a risk factor in Asians and a protective factor in Caucasians under all four genetic models; however, these associations were not statistically significant. This finding may have happened by chance, or may have resulted from different gene frequencies in the different populations; the MAF of XRCC1 Arg194Trp is 0.26 for Asians but only 0.09 for Caucasians. A number of studies have reported the association between the XRCC1 Arg194Trp polymorphism and oral cancer risk [10], [39], [41], [43], [44], [51]. It has been reported that the XRCC1 Arg194Trp polymorphism may result in decreased repair efficiency of DNA damage, and the repair deficit may eventually increase an individual's susceptibility to oral cancer [53], [54]. In our subgroup meta-analysis, we also detected the association of XRCC1 Arg194Trp with oral cancer risk. We found that under the allelic model. Trp allele carriers had a higher risk of oral cancer than Arg allele carriers. We also found that individuals with the Arg/Trp genotype had a higher risk of developing oral cancer under the heterozygote model; however, this association was not detected under the homozygous model. We speculate that the main reason for this finding may be the low occurrence of the Trp/Trp genotype in the study populations; indeed, in several studies, the number of Trp/Trp genotype was reported to be zero. The low occurrence of the Trp/Trp genotype will lead to poor statistical power. Under the dominant model, when the Trp/Trp and Arg/Trp genotypes were analyzed together, the association of the Arg/Trp genotype with oral cancer was still statistically significant. This finding is in accordance with our speculation that the heterozygote and homozygote models gave different results because of the low occurrence of the Trp/Trp genotype in the study population. However, this hypothesis needs to be tested with larger sample sizes in future studies.

The XRCC1 Arg399Gln polymorphism is located in the zinc finger domain area (PARP binding site) of the protein that detects DNA strand breaks [55]. The carriers of this variant were shown to have a higher level of DNA adducts [56] and tobacco-related DNA damage [46], [57]–[59]. XRCC1 Arg399Gln has been reported to be significantly associated with risks of gastric [60], lung [61], and colorectal [20] cancers. Ramachandran et al. found that the XRCC1 Arg399Gln polymorphism was associated with increased risk of oral cancer in an Indian population [10], while Kostrzewska-Poczekaj et al. found that XRCC1 Arg399Gln was a protective factor for squamous cell carcinoma of the head and neck in young adults [52]. Most other studies have found no significant association of XRCC1 Arg399Gln with HNC risk [9], [25], [34], . In the present study, we also found no association between XRCC1 Arg399Gln and HNC risk under all four genetic models.

The XRCC1 Arg280His polymorphism is located in the proliferating cell nuclear antigen binding region [62] in the apurinic/apyrimidinic endonuclease (APE)-binding domain of the protein [54], [63]. The Arg280His polymorphism could potentially alter the structure of XRCC1 and affect its ability to interact with APE [54], [64]. In a functional study, the XRCC1 protein carrying His 280 failed to rescue the single-strand break repair deficiency of mutant cells when human XRCC1 variant proteins were introduced into XRCC1 mutant Chinese hamster ovary cells [65]. Although functional studies revealed a possible mechanism for the association of the XRCC1 Arg280His polymorphism with cancer risk, our meta-analysis did not detect a significant association between XRCC1 Arg280His and HNC risk. This null result may be because of the limited number of studies that were included in our analyses. Clearly, larger sample sizes are needed to clarify the association of the XRCC1 Arg280His polymorphism with HNC risk.

Although we conducted a comprehensive analysis, our study has a number of limitations. First, only a limited number of eligible studies were found and so the sample size was relatively small. Therefore, especially in the stratified analyses, the association detected in our study may have occurred by chance. Second, because almost all the studies that were selected for meta-analysis were case-control studies, the patients were cancer survivors and patients who did not survive were not included. As a result, selection/survival bias could not be avoided.

In conclusion, the meta-analysis detected no association between the XRCC1 Arg399Gln and Arg280His polymorphisms and risk of HNC. However, in the subgroup analyses of studies adjusted for smoking and alcohol, the XRCC1 Arg194Trp polymorphism was associated with increased risk of HNC and, in the stratified analyses based on cancer site, XRCC1 Arg194Trp was associated with increased risk of oral cancer. Further studies with larger samples are needed to further evaluate the association between XRCC1 polymorphisms and HNC risk.

## Supporting Information

Figure S1 **Sensitivity analysis of XRCC1 Arg194Trp using the allelic model.**
(DOC)

Figure S2 **Begg's funnel plot of publication bias test for XRCC1 Arg399Gln using the dominant model.**
(DOC)

Supplement S1 **PRISMA Checklist.**
(DOC)

Supplement S2 **PRISMA Flowchart.**
(DOC)
