# Supplementary figures and images for: Association of X-Ray Repair Cross-Complementing Group 1 Arg194Trp, Arg399Gln and Arg280His Polymorphisms with Head and Neck Cancer Susceptibility: A Meta-Analysis
Source: PLoS One. 2014 Jan 30;9(1):e86798. doi: 10.1371/journal.pone.0086798 (PMC3907446; doi:10.1371/journal.pone.0086798)

Figure S1: Sensitivity analysis of XRCC1 Arg194Trp using the allelic model.

**
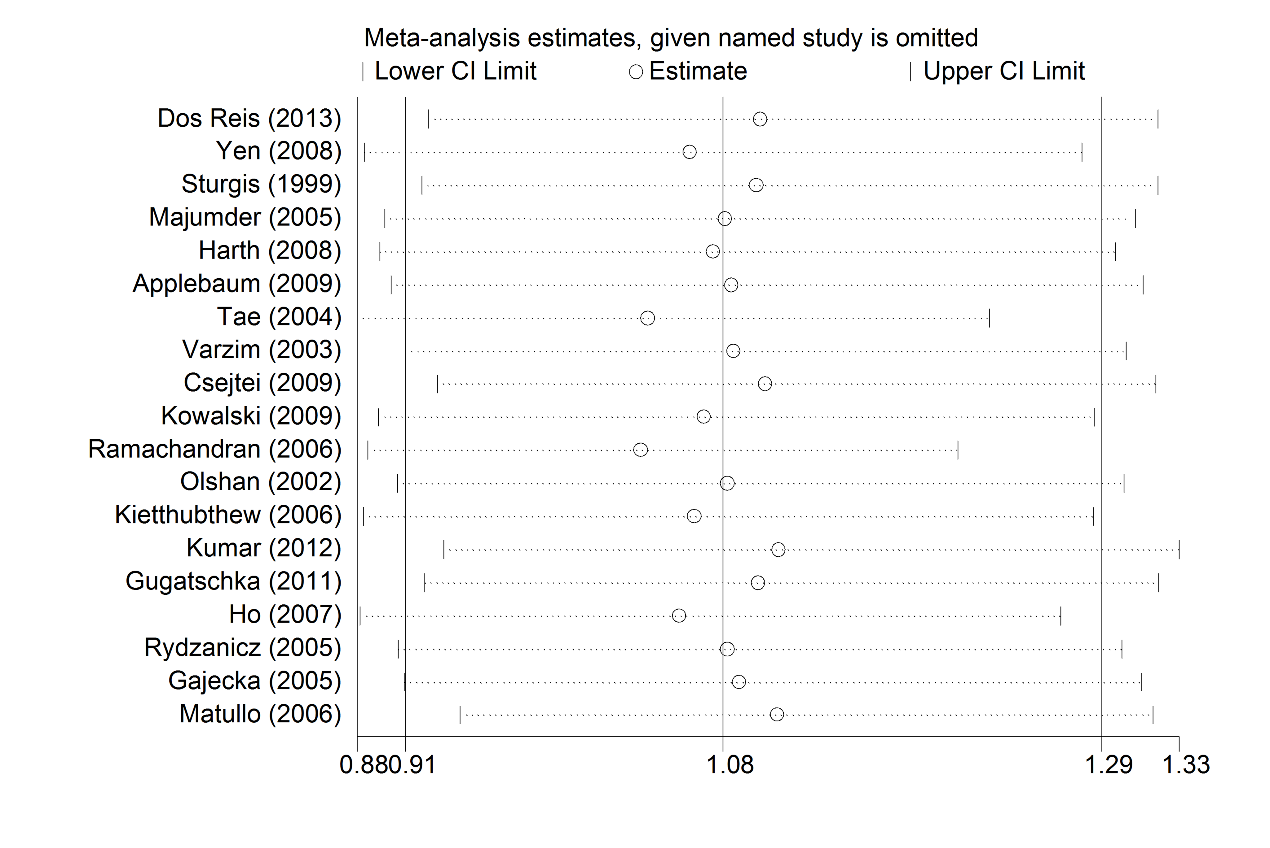
**

Supplement: Figure S1 — Sensitivity analysis of XRCC1 Arg194Trp using the allelic model. (DOC) [file pone.0086798.s001.doc]

Figure S2: Begg’s funnel plot of publication bias test for XRCC1 Arg399Gln using the dominant model.

**
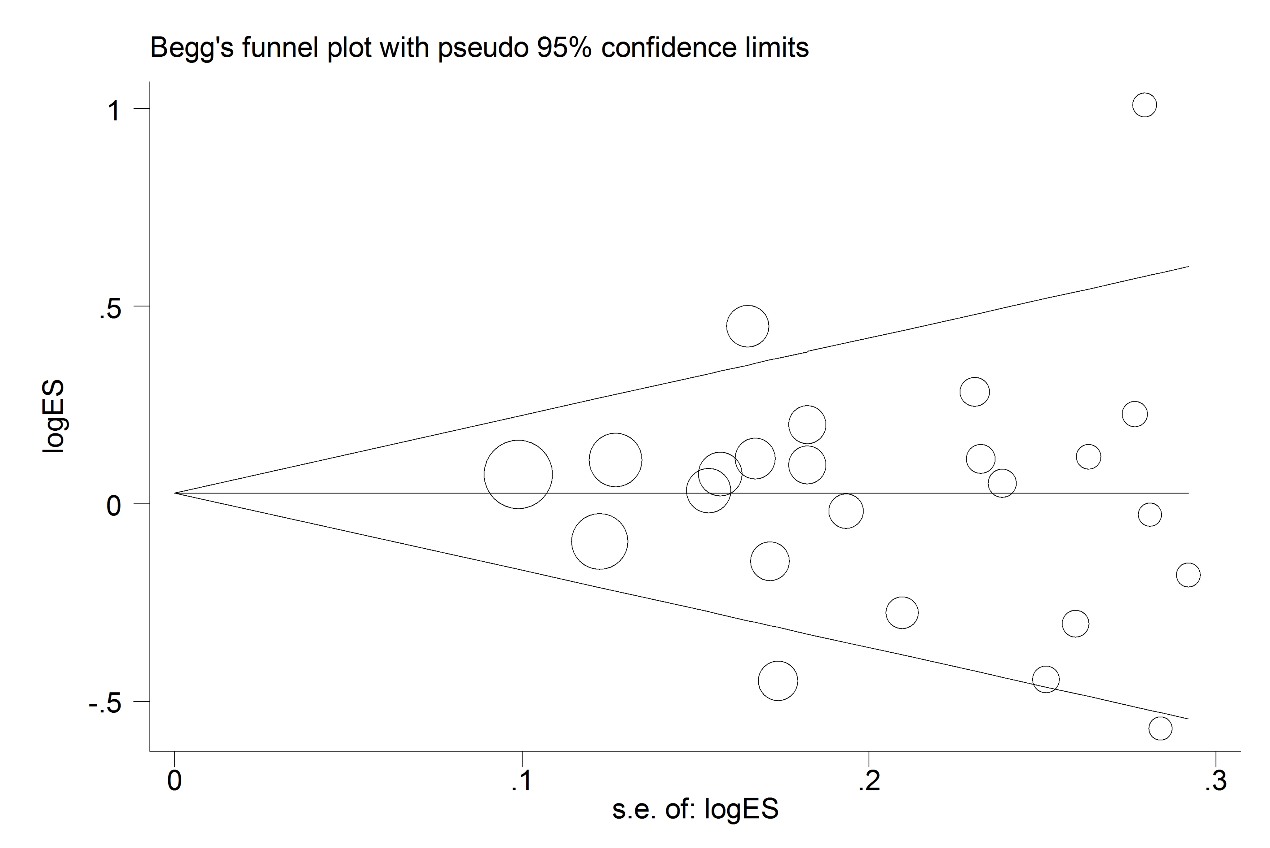
**

Supplement: Figure S2 — Begg's funnel plot of publication bias test for XRCC1 Arg399Gln using the dominant model. (DOC) [file pone.0086798.s002.doc]
