# Supplementary material for: Association of X-Ray Repair Cross-Complementing Group 1 Arg194Trp, Arg399Gln and Arg280His Polymorphisms with Head and Neck Cancer Susceptibility: A Meta-Analysis
Source: PLoS One. 2014 Jan 30;9(1):e86798. doi: 10.1371/journal.pone.0086798 (PMC3907446; doi:10.1371/journal.pone.0086798)
Supplement: Supplement S2 — PRISMA Flowchart. (DOC) [file pone.0086798.s004.doc]

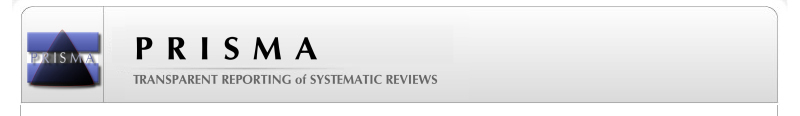
**PRISMA 2009 Flow Diagram**

**Screening**

**Included**

**Eligibility**

**Identification**

Records identified through database searching
(n = 224)

Additional records identified through other sources
(n = 0)

Records after duplicates removed
(n = 168)

Records screened
(n = 168)

Records excluded
(n = 125)

Full-text articles assessed for eligibility
(n = 43)

Full-text articles excluded, with reasons
(n = 17)

Studies included in qualitative synthesis
(n = 26)

Studies included in quantitative synthesis (meta-analysis)
(n = 26)
